# Case of Strangulated Inguinal Hernia, Occurring Unexpectedly in an Old Ruptured Subject

**Published:** 1861-03

**Authors:** Thomas S. Duffy

**Affiliations:** Rutherfordton, N. C.


*Case of Strangulated Inguinal Hernia, occurring unexpectedly in an old ruptured subject.* By Thomas S. Duffy, M. D. of Rutherfordton, N. C.

August 3d.—Daniel Lane, a resident of Lincoln county, had been for many years past ruptured on the *left* side. On this day, directly after breakfast, he took leave of his father-in-law, and mounted his horse to ride home. He had only proceeded a few yards when for *the first time* the bowel escaped through the *right* inguinal ring and became strangulated. The pain was so great he had to be carried into the house. On the night of the same day I was called on to visit him and arrived to his assistance at day-light, next morning—distance twenty miles. The large size, and extreme tenseness of the tumor, gave little hope of its reduction. When the taxis had failed, his situation and chances were explained to him, and he expressed himself as anxious for the operation. This was at once performed, the country people who had crowded in giving ready assistance. The hernial coverings were thin. The sac contained some muddy, yellow fluid, with about five inches of colon of a dark chocolate color. The stricture was narrow and the fibres of the tendon, when cut, flew apart with violence. The bowel could not be returned, on account of partial adhesion, till an additional portion was drawn out, when there was no further difficulty. The wound was now adjusted with sutures and compresses, which were kept moist with tepid water.

Two hours after the operation the pulse was full, with uneasy abdominal feelings. I bled him to the extent of a pint or more, and gave 10 grains of calomel with half a dram of tincture of opium.

At 4 o'clock P. M., he was resting comfortably. I left a mixture composed of morphine and compound spirit of ammonia—a tablespoonful to be given at one time, and repeated as often as necessary to relieve pain or restlessness. Half an ounce of castor oil to be given next morning.

On the 7th, I visited him and dressed the wound. His bowels had acted. I repeated the anodyne mixture and left three 5-grain papers of calomel—one to be taken every night.

On the 13th I visited him again. The wound had sicatrised. The gums were slightly touched. I found an abscess in the integument—caused probably by the efforts at reduction—which on being lanced yielded laudable pus. From this time the patient rapidly recovered.

